# Deciphering the Intercellular Communication Network of Peripartum Decidua that Orchestrates Delivery

**DOI:** 10.3389/fcell.2021.770621

**Published:** 2021-11-05

**Authors:** Jingrui Huang, Weishe Zhang, Yanhua Zhao, Jingzhi Li, Mingkun Xie, Yang Lu, Qiaozhen Peng, Jiejie Zhang, Ping Li, Lei Dai

**Affiliations:** ^1^ Department of Obstetrics, Xiangya Hospital Central South University, Changsha, China; ^2^ Hunan Engineering Research Center of Early Life Development and Disease Prevention, Changsha, China

**Keywords:** decidua, delivery, intercellular communication, B cell receptor repertoire, T cell receptor repertoire, single-cell RNA sequencing

## Abstract

Intercellular communication in the decidua plays important roles in relaying information between the maternal and fetal systems in the maintenance of pregnancy and the transition to labor. To date, several studies have explored cell-cell communications in the decidua during different periods of pregnancy, but studies systematically decoding the intercellular communication network, its internal cascades, and their involvement in labor are still lacking. In this study, we reconstructed a decidual cell-cell communication network based on scRNA-seq of peripartum decidua via the CellCall method. The results showed that endometrial cells (EECs) and extravillous trophoblasts relayed most of the common intercellular signals in the decidua both before delivery (DBD) and after delivery (DAD). Endothelial cells and EECs controlled many WNT-signaling-related intercellular communication factors that differed between DBD and DAD, some of which could be candidate biomarkers for the diagnosis of labor. Analysis of intercellular communications related to T cells identified abundant maternal-fetal immune-tolerance-related communication, such as TNFSF14-TNFRSF14/LTBR and FASLG-FAS signalings. We further explored the characteristics of the B cell receptor (BCR) and T cell receptor (TCR) repertoires by single-cell BCR/TCR sequencing. The results showed no significant differences in clonal expansion of B/T cells between DAD and DBD, indicating there was no significant change to adaptive immunity at the maternal-fetal interface during delivery. In summary, the findings provide a comprehensive view of the intercellular communication landscape in the peripartum decidua and identified some key intercellular communications involved in labor and maternal-fetal immune tolerance. We believe that our study provides valuable clues for understanding the mechanisms of pregnancy and provides possible diagnostic strategies for the onset of labor.

## 1 Introduction

Labor is triggered by a series of complex communications via fetal and maternal factors that act upon the uterus to trigger intercellular pathways, leading gradually to coordinated cervical ripening and myometrial contractility (Kniss and Iams, 1998). However, the exact mechanisms and communication cascades involved in delivery remain uncertain. The maternal-fetal interface, which is composed of cells of both maternal and fetal origin, is a key heterogeneous organ connecting the maternal and fetal systems during pregnancy and plays key roles in delivery (Ishida et al., 2011; Maltepe and Fisher, 2015). The decidua, an important tissue found within the maternal-fetal interface, is the site of the intercellular crosstalk that plays important roles in connecting the maternal and fetal systems, the maintenance of pregnancy, and the transition to labor (Areia et al., 2017). Despite advances in technology, our understanding of the highly integrated and extremely dynamic nature of the decidua and its functions during pregnancy and labor is still far from clear (Mori et al., 2016).

Recently, with the rapid development of single-cell RNA sequencing (scRNA-seq) technologies, many research groups have begun to investigate the cellular composition of the maternal-fetal interface and/or decidua at different stages of pregnancy and have obtained a comprehensive understanding of the cellular organization, homeostasis, dynamics, and differentiation of the placenta (Tsang et al., 2017; Liu et al., 2018; Suryawanshi et al., 2018; Vento-Tormo et al., 2018). For example, Vento-Tormo et al. (Vento-Tormo et al., 2018) developed an atlas of the first-trimester human placenta by scRNA-seq and identified an array of cell types unique to the early maternal-fetal interface. Moreover, scRNA-seq of sorted placental cells from first- and second-trimester human placentae identified several new trophoblast subtypes and human placental trophoblast differentiation during the early stage of pregnancy (Liu et al., 2018). In our previous study, we characterized the single-cell landscape of the peripartum decidua, identified the major cell populations and subpopulations of the decidua, and revealed decidual cell changes during labor (Huang et al., 2021). These cell atlas studies have provided important resources for future explorations of pregnancy and its complications (Rajagopalan and Long, 2018).

To data, there are few specialized histomorphological studies on decidua during peripartumperiod. Elfayomy and Almasry (2014) has evaluated the histomorphology of the peripartum fetal membranes and found that the apoptotic bodies differentially expressed in different cell types, and the proinflammatory cytokines including tumor necrosis factor-alpha and vascular endothelial growth factor significantly increased with onset of labor. Osman et al. (2006) has reported that all regions of fetal membrane and decidua contribute to the inflammatory process of human parturition. After labor onset, the decidual tissue may have different histomorphological changes which are related to various cell types and cytokines. Therefore, the study of interplay among various decidual cells and the cytokines would be helpful for a more detailed evaluation for decidua and delivery.

The decidua mediates communication between two semiallogenic individuals, the mother and the fetus, which is the epitome of intercellular communication (Iliodromiti et al., 2012; Pavličev et al., 2017). Therefore, the further elucidation of the intercellular communication in the decidua could facilitate our understanding of the fundamental basis of pregnancy and help to reveal pathogenic mechanisms of pregnancy-related disorders (Zhang et al., 2021b; Chen et al., 2021). Intercellular communication network analysis using scRNA-seq of human term placenta has found that the decidua is the center of intercellular signal transduction and has indicated the dominant role of growth factors and immune signals in the intercellular crosstalk (Tsang et al., 2017). Cell-cell communication analysis of first-trimester placentas identified many regulatory interactions that prevent harmful innate or adaptive immune responses in the maternal-fetal interface environment (Vento-Tormo et al., 2018). Suryawanshi et al. (2018) also reported many putative intercellular communications in the fetal-maternal microenvironment. However, with the exception of the above studies, research attempting to systematically decode the network and internal cascades of the cell-cell communication involved in labor is still lacking.

In this study, we aimed to visualize the intercellular communication that occurs during the perinatal period and identify key intercellular transduction signaling pathways related to the onset of labor. We collated the scRNA-seq data for term decidua before delivery (DBD) and after delivery (DAD) obtained in our previous study (Huang et al., 2021) and reconstructed the cell-cell crosstalk via the CellCall method (Zhang et al., 2021a). We then investigated the shared and differential intercellular signals between DBD and DAD and explored the intercellular crosstalk between T cells and other decidual cells. Lastly, to reveal the dynamic changes in the maternal-fetal immune system during delivery, we further explored the characteristics of the B cell receptor (BCR) and T cell receptor (TCR) repertoire between DBD and DAD through single-cell BCR/TCR sequencing (scBCR/TCR seq).

## 2 Materials and Methods

### 2.1 scRNA-Seq Data Collection

The processed scRNA-seq data of 29,231 peripartum decidual cells from our previous study were collated (including 17,149 DBD cells and 12,082 DAD cells) (Huang et al., 2021). There were eight main types of peripartum cells, including 10,004 endothelial cells (ECs), 6,422 decidual stromal cells (DSCs), 5,277 extravillous trophoblasts (EVTs), 1,194 T cells (TCs), 3,720 smooth muscle cells (SMCs), 1,312 Dendritic cells (DCs), 1,133 fibroblasts (FBs), and 169 endometrial cells (EECs). The gene expression levels were normalized by log2 [TPM/10 + 1] (transcripts per million, TPM).

### 2.2 Inferring Cell-Cell Communication by CellCall

Intercellular and internal signaling among different cell types of the decidua was inferred by CellCall (Zhang et al., 2021a), which is a toolkit for researching intercellular communication networks and internal regulatory signals by combining the expression of ligands/receptors with downstream transcription factor (TF) activities for certain ligand-receptor (L-R) pairs. The technique also has an embedded pathway-activity analysis method to help explore the main pathways involved in communication between certain cells. Genes that were expressed in less than 10% of the cells of a certain cell type were excluded in this study.

### 2.3 Ethics Statement, Informed Consent, and Sample Preparation

A total of six peripartum decidua samples (three DBD samples and three DAD samples) were obtained from Xiangya Hospital Central South University or Changsha Hospital for Maternal and Child Health Care. Informed consent was obtained from all patients prior to data collection, which are the same samples as our previous study (Huang et al., 2021). The processing of tissue dissociation can be seen in our previous report (Huang et al., 2021). Then the cell suspension was prepared for the scRNA-seq cDNA library and scBCR/TCR cDNA library preparation and sequencing, respectively. The study protocol was approved by the Medical Ethics Committee of the Xiangya Hospital Central South University (2018081027) and Changsha Hospital for Maternal and Child Health Care Ethics Committee (2018810).

### 2.4 scBCR-Seq and Analysis

Full-length BCR V(D)J segments were enriched from cDNA amplified from 5′ libraries using a Chromium Single-Cell V(D)J Enrichment kit in accordance with the manufacturer’s protocol. BCR sequences for each single B cell were assembled by Cell Ranger vdj pipeline (v.3.0.2). Only those cells with both productive immunoglobulin heavy chains (IGH) and productive immunoglobulin light chains kappa (IGK) or lambda (IGL) were kept. If more than one heavy chain or light chain was detected in a single cell, the cell with the chain with the highest amount of unique molecular identifiers (UMI) was retained (Zheng et al., 2017). A clonotype was defined as a unique pairwise combination of IGH/IGK/IGL. A cell was considered to be clonally expanded if its clonotype was shared by at least two cells. The clonality of a clonotype was indicated by the number of cells with the same clonotype (performed by CapitalBio Technology, Beijing). Based on the scBCR-seq data, a total of 8,755 B cells were detected.

### 2.5 scTCR-Seq and Analysis

VDJ segments were generated using the Chromium Single-Cell V(D)J Enrichment kit following the manufacturer’s protocol. The Cell Ranger vdj pipeline was applied to assemble the TCR sequences and identify the CDR3 sequence and TCR genes. Then, the cells were filtered according to the following steps: 1) Cells annotated as T cell clusters in scRNA-seq were kept, and 2) cells that possessed productive TCR α and β chains were incorporated into the analysis. If more than one α or β chain was detected in a cell, we retained the chain with the highest UMIs (Zheng et al., 2017). We defined the expanded clonal cells as those having a pair of TCR α and β chains that appeared in at least two cells. (performed by CapitalBio Technology, Beijing). Based on the scTCR-seq data,a total of 4,745 T cells were detected.

### 2.6 Clonal Diversity and Evenness Analysis

The clonal diversity of BCR/TCR was estimated by Shannon Entropy and the D50 index (Al Khabouri et al., 2021). Shannon entropy (H) estimated both richness (number of clonotypes) and diversity (evenness of distribution) (Sims et al., 2016). The formula used was as follows:

$$H_{\mathrm{clonotypes}}=H_{\mathrm{VJ}}+H_{\Delta }$$

where *H*_*VJ*_ is the entropy of the distribution of VJ cassette combinations, and *H*_*Δ*_ is the entropy from the VJ-independent component. D50 is defined as the smallest percentage of different clonotypes that make up at least half of the total clonotypes in a population or subpopulation of B/T cells (Wang et al., 2021). The clonal evenness of BCR/TCR was estimated by the Gini coefficient.

## 3 Results

### 3.1 General Picture of Intercellular Communication in Peripartum Decidua

**FIGURE 1:**
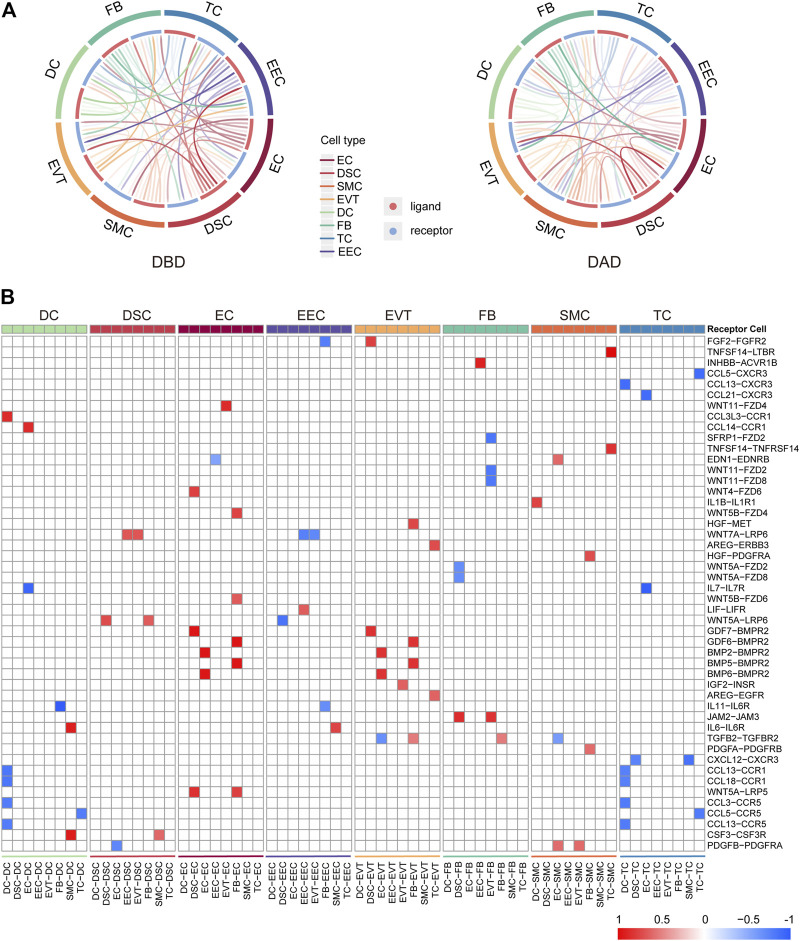
General picture of intercellular communication in peripartum decidua. **(A)** Circos plot of intercellular communication among different cell types in DBD and DAD. **(B)** Heatmap of differential intercellular signals between DBD and DAD; red represents significantly increased intercellular communication in DAD; blue represents significantly decreased intercellular communication in DAD.

Initially, to comprehensively investigate the crosstalk in the peripartum decidua during delivery, we investigated the intercellular communications among the eight cell types of the decidua by CellCall. As shown in Figure 1A, various intercellular communication signals were widely distributed among the different cell types in both DBD and DAD. Compared to the other cell types, EECs and DSCs relayed significantly more signals to and from other cells, indicating the dominant role of EECs and DSCs in the intercellular crosstalk in the maternal-fetal interface during delivery. We further investigated the differential intercellular signals between DBD and DAD (Figure 1B) and found that the differential signals relating to ECs and EVTs were significantly increased after delivery and were mainly involved in BMP-BMPR signaling. In contrast, the differential intercellular communications related to EECs and two immune cells (TCs and DCs) were significantly decreased after delivery and were mainly involved in CCL-CCR signalings.

### 3.2 Common Intercellular Communications in DBD and DAD

**FIGURE 2:**
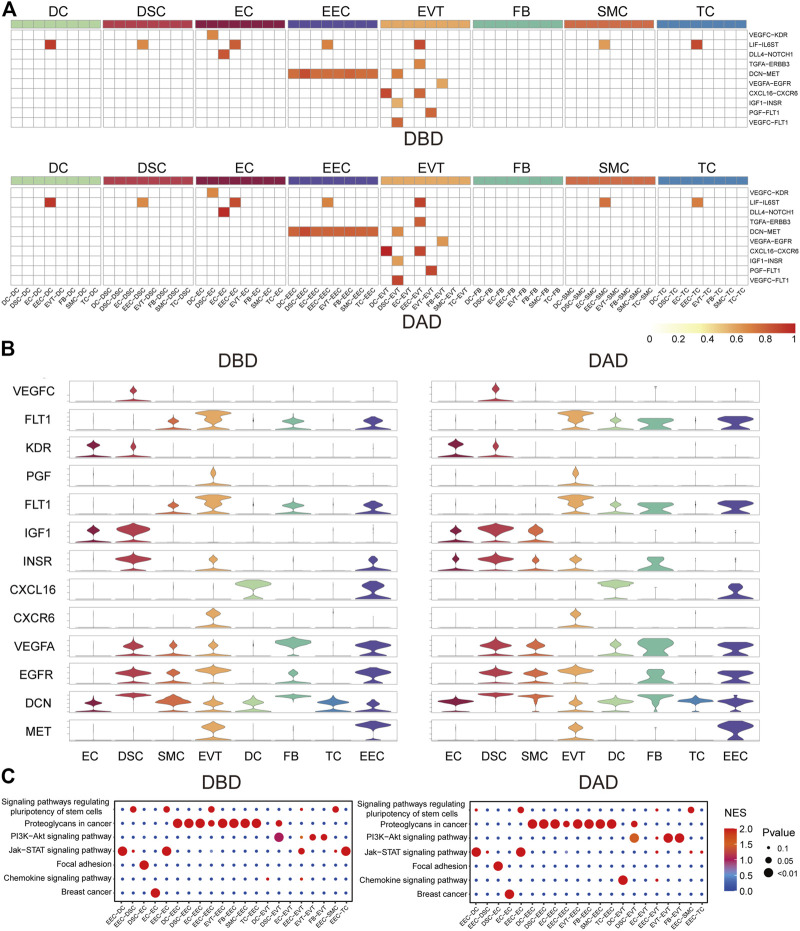
Common forms of intercellular communications in DBD and DAD. **(A)** Heatmap of common intercellular signals among different cell types in DBD and DAD; red represents the score of intercellular communication. **(B)** Expression of ligands and receptors of common intercellular signals. **(C)** Pathway activity analysis of common intercellular communication in DBD and DAD.

To investigate the essential intercellular signals during delivery, we identified the intercellular communications (with a score larger than 0.5) common to both DBD and DAD. As shown in Figures 2A,B, compared to other cells, EECs and EVTs relayed more common intercellular communications. The key common intercellular communication between EECs and other cells was DCN-MET signaling. DCN is an important molecule for maintaining the homeostatic balance between the naturally invasive human placenta and the maternal uterus in pregnancy (Lala and Nandi, 2016); its actions at the fetal-maternal interface include the restraint of trophoblast migration and invasion and uterine angiogenesis by binding to multiple TKRs, including MET (Chen, 2014). In EVTs, LIF- LIFR (including IL6ST) signaling has been shown to play an important role in trophoblast invasion *in vivo* and may facilitate trophoblast decidual immune cell crosstalk to enable adequate spiral artery remodeling (Winship et al., 2015). CXCL16/CXCR6 interaction promotes endometrial decidualization via the PI3K/AKT pathway (Mei et al., 2019). PGF/VEGFC-FLT1 signals have also been demonstrated to enhance embryo development, improve endometrial receptivity, and facilitate interactions between the developing embryo and the endometrium (Guo X. et al., 2021). Moreover, pathway activity analysis showed that these common intercellular communications were mainly enriched in the PI3K-Akt signaling pathway, Jak-STAT signaling pathway, focal adhesion, and proteoglycans in cancer (Figure 2C). These pathways have been reported to be critical for the implantation, decidualization, and aging of the placenta (Gupta et al., 2016; Menon, 2016; Sharma et al., 2016).

### 3.3 Differential Intercellular Communication Between DAD and DBD

**FIGURE 3:**
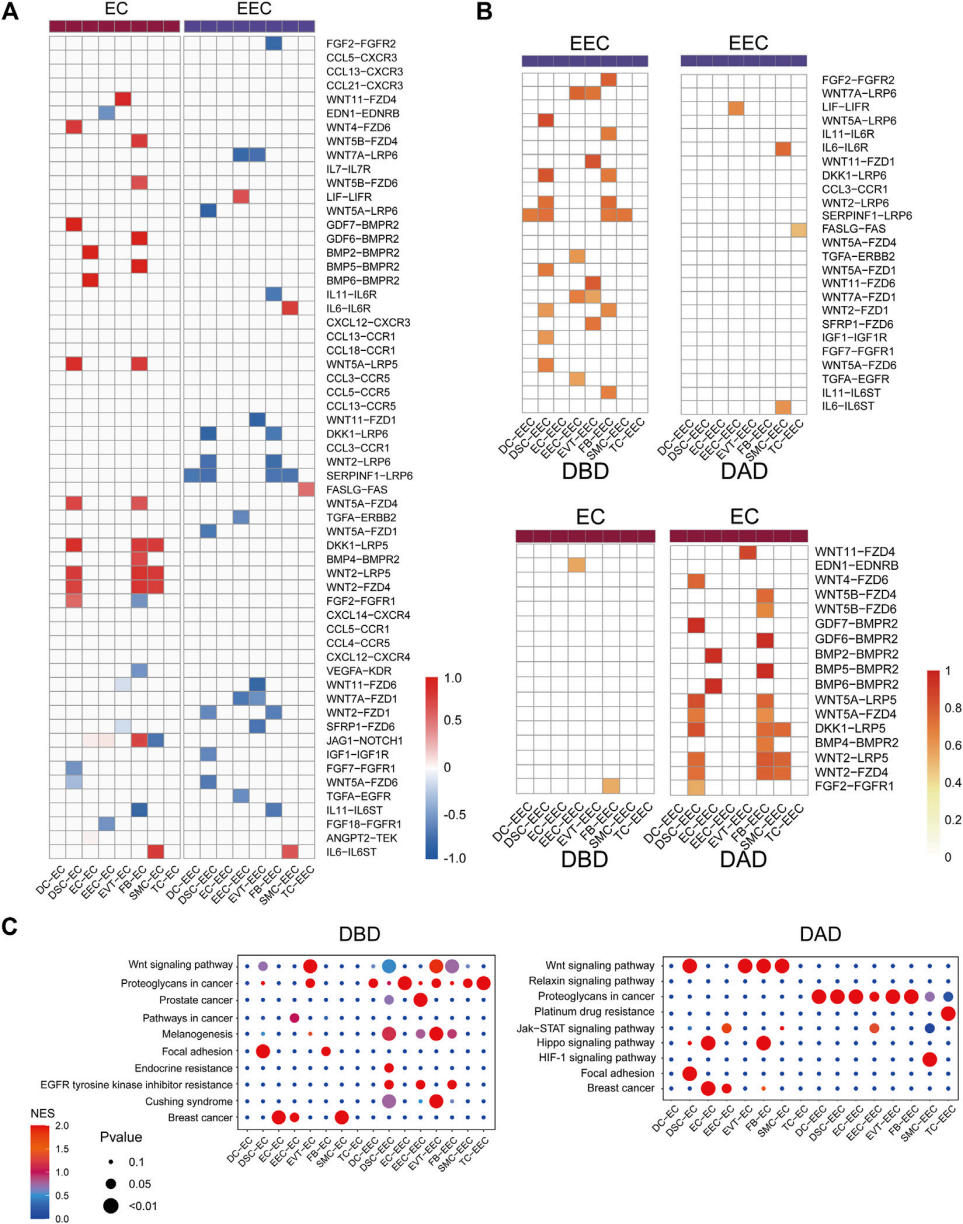
Differential intercellular communication between DAD and DBD. **(A)** Heatmap of differential intercellular signals related to ECs and EECs; red represents significantly increased intercellular communication in DAD; blue represents significantly decreased intercellular communication in DAD. **(B)** Details of differential intercellular communication related to ECs and EECs; red represents the score of intercellular communication. **(C)** Pathway activity analysis of differential intercellular communication related to ECs and EECs.

We further investigated the differential intercellular communications between the DBD and DAD, and found many of these signals were relayed by the two representative decidual tissue cells ECs and EECs (Figure 3A). Many distinct intercellular signals in the DAD were related to communication between EC and other cells (Figure 3B). For example, many WNT signals have been identified as being relayed by ECs, which are very important signaling cells in implantation and decidualization, and changes in Wnt signaling components have been recorded in cancers of reproductive tissues, endometriosis, and gestational diseases (Zhang and Yan, 2016). BMPs-BMPRs have also been proven to regulate uterine decidualization via the Wnt signaling pathway (Li et al., 2007). Pathway activity analysis showed that these differential intercellular communications were mainly enriched in the Wnt signaling pathway, Hippo signaling pathway, and focal adhesion, among others (Figure 3C). In contrast to ECs, numerous differential intercellular communications between EECs and other cells occurred in the DBD, in which WNT signals also play an important role in intercellular crosstalk between EEC and other cells. Pathway activity analysis showed that the expression of intercellular communications was enriched mainly in the Wnt signaling pathway, proteoglycans in cancer, and prostate cancer etc. (Figure 3C).

### 3.4 Differential Intercellular Communication Related to TCs

**FIGURE 4:**
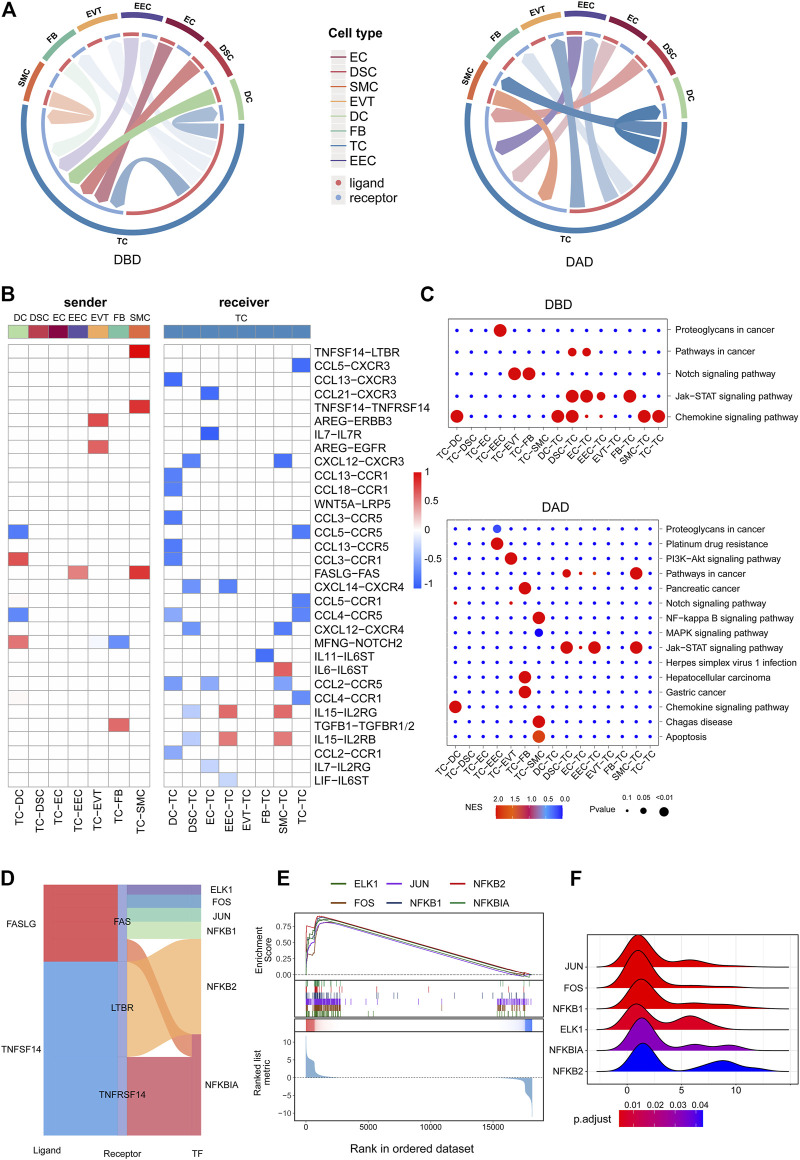
Intercellular communication related to TCs. **(A)** Circos plot of intercellular communication related to TCs in DBD and DAD. **(B)** Differential intercellular communication related to TCs; red represents significantly increased intercellular communication in DAD; blue represents significantly decreased intercellular communication in DAD. **(C)** Pathway activity analysis of differential intercellular communication related to TCs. **(D)** Sankey plot of three intercellular signals related to TCs. **(E)** Enrichment analysis of six TFs (the target gene set of the TF) downstream of the three intercellular signal pathways. **(F)** Ridge plot of the density distribution of FC of TGs for the six TFs.

Contemporary studies have shown that T cells play key roles in the decidua during human pregnancy (Powell et al., 2017). Hence, we further investigated the aspects of intercellular communication between TCs and other decidual cells. As shown in Figures 4A,B, compared to the DBD, intercellular signals from TCs to other cells were obviously increased in the DAD. Contrastingly, the intercellular communication between TCs and other cells obviously decreased. Some of the differential intercellular signals between TCs and other cells in the DAD are reportedly related to the physiological and/or pathological processes of pregnancy, of which TNFSF14-TNFRSF14/LTBR signals have been reported to be significantly increased in patients with recurrent pregnancy loss (Guo C. et al., 2021). FASLG-FAS signaling from TCs to the decidua was demonstrated to be related to mother-fetal immune tolerance (Guller and Lachapelle, 1999). Pathway activity analysis showed that these differential intercellular communications were mainly enriched in the Notch signaling pathway and PI3K/AKT pathway, etc. (Figure 4C). Further analysis of the TFs downstream of the TNFSF14-TNFRSF14/LTBR and FASLG-FAS pathways revealed that most were involved in immune tolerance in pregnancy (Figure 4D) (Rackaityte and Halkias, 2020; Gómez-Chávez et al., 2021). For example, NFκB family members NFKB1, NFKB2, and NFKBIA occupy central roles in the immune microenvironment (Rackaityte and Halkias, 2020; Gómez-Chávez et al., 2021). Enrichment analysis indicated that all these TFs were distinctly activated (see Figure 4E), and most target genes (TGs) had fold change (FC) values greater than 1 (Figure 4F). Furthermore, most of the intercellular communications between TCs and cells in the DBD are also reportedly related to pregnancy (Mincheva-Nilsson et al., 2000). Studies have reported that various decidua cells, such as EVTs, DSCs, and EECs, regulate mother-fetal immune tolerance and the microenvironment by targeting T cells via the chemokine network (Ramhorst et al., 2016). Pathway activity analysis revealed that these intercellular signals were mainly enriched in the chemokine signaling and Jak-STAT signaling pathways, etc. (Figure 4C).

### 3.5 scBCR/TCR-Seq Profiling of Decidua

**FIGURE 5:**
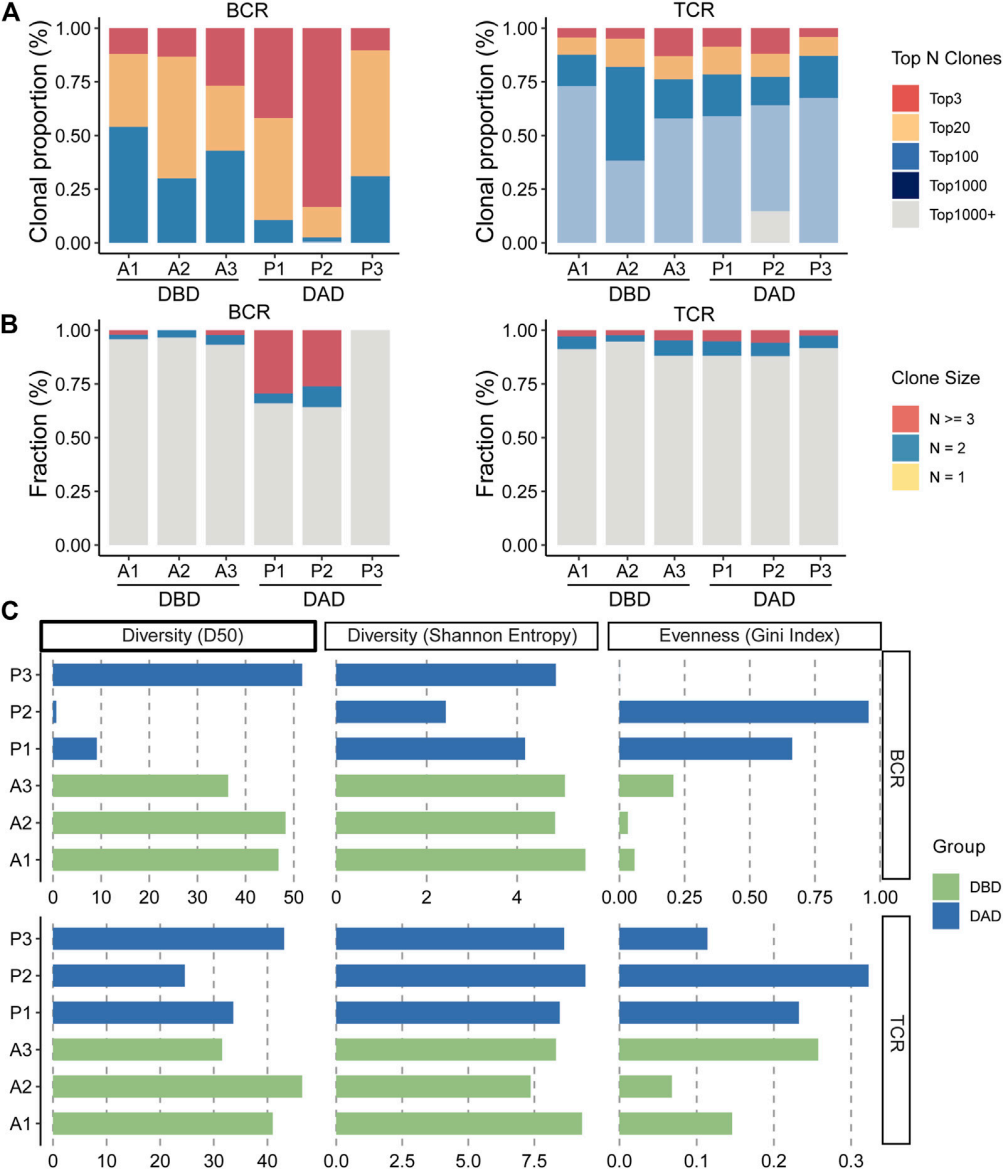
Characterization of BCR/TCR repertoires in six decidua samples. **(A)** Proportion of the top N most frequently occurring clones. **(B)** Proportion of unique and non-unique BCR/TCR clones. **(C)** D50, Shannon entropy, and Gini-coefficient scores of the BCR/TCR clones in six samples.

**FIGURE 6:**
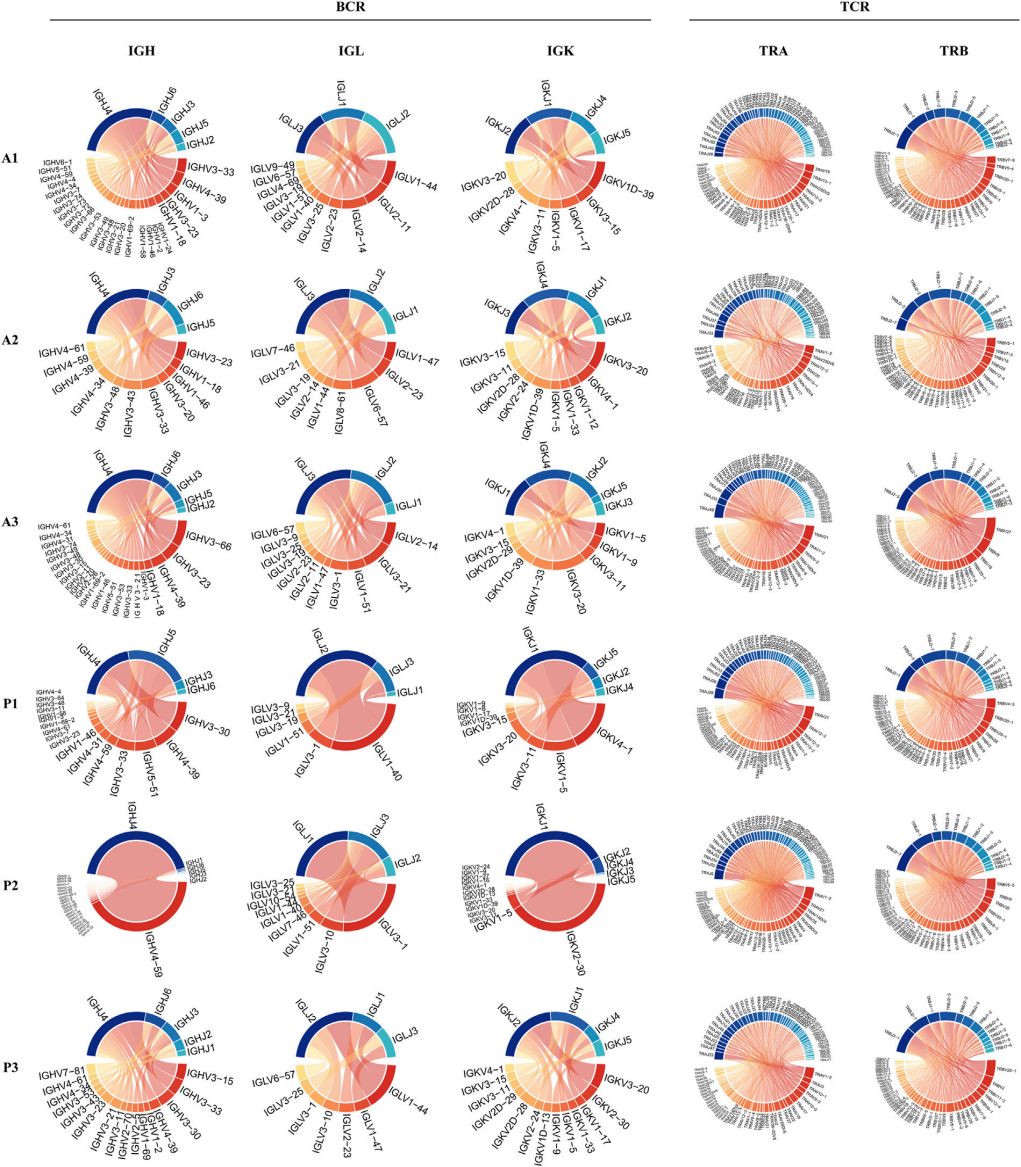
Circos plots of IGH, IGL, and IGK recombinations of BCRs and TRA and TRB recombinations of TCRs.

To reveal the dynamic changes that occur in the maternal-fetal immune system during delivery, we further explored the characteristics of the BCR and TCR repertoires in the DBD and DAD using scBCR/TCR seq. As shown in Figures 5A,B, the frequencies of clonal B cells and clonal T cells seemed to be higher in the DAD than the DBD. However, the Shannon Entropy and D50 indexes of the six samples suggested that the clonal diversity of BCR/TCR did not markedly change between the DBD and DAD (Figure 5C). The Gini coefficient across the samples indicated that the clonal evenness of BCR/TCR also did not markedly change between the DBD and DAD. Moreover, the top IGH, IGL, and IGK recombinations of BCRs were often observed in a large percentage of DBD samples (Figure 6), but the T cell receptor α (TRA) and T cell receptor β (TRB) recombinations of TCRs did not differ remarkably between the DAD and DBD (Figure 6).

## 4 Discussion

The decidua is a key intrauterine source of bioactive molecules that are pivotal in pregnancy and parturition and crucial to the crosstalk between maternal and fetal compartments (Liu et al., 2003; Pavličev et al., 2017; Yang et al., 2019). Therefore, decoding the intercellular signaling network in the decidua involved in the onset of labor could not only help to elucidate the exact mechanisms of labor but also reveal candidate biomarkers for the diagnosis of labor onset. Our previous study revealed the communication landscape in the decidua before and after delivery (Huang et al., 2021), but the similarities and differences in the intercellular signals involved in delivery have not been fully characterized (Zhao et al., 2019). Here, we comprehensively analyzed an intercellular communication network involving multiple cell types in the peripartum decidua and found that EECs and DSCs sent and received significantly more signals than other cells. The communication lines related to ECs and EVTs were significantly increased after delivery, and the signals relayed to EECs and two immune cells (TCs and DCs) were significantly decreased after delivery. We further investigated the TC-related communications between the DBD and DAD, and the findings indicated that TCs play key roles in full-term delivery. Finally, the results of scTCR/BCR-seq showed no significant differences in the clonal expansion of B/T cells between the DAD and DBD, which indicated there were no significant changes to adaptive immunity at the maternal-fetal interface during delivery.

When we investigated the intercellular communication common to both the DBD and DAD, we found that EECs and EVTs were prominent receivers of signals from other cells. DSCs constitute the main cellular component of human decidua and show activities that appear to play important roles in embryo implantation, the development of pregnancy, and maternal-fetal immune tolerance (Macklon and Brosens, 2014; Muñoz-Fernández et al., 2019). EVTs at the end of the placental villi invade and implant into the maternal decidua, establishing critical tissue connections at the maternal-fetal interface (Chen et al., 2009). The communications related to these cells, such as DCN-MET, LIF-LIFR, and CXCL16/CXCR6 signaling, have exhibited key roles in the maintenance and development of pregnancy (Chen, 2014; Winship et al., 2015; Mei et al., 2019), and the enriched pathways, such as PI3K-Akt signaling pathway, Jak-STAT signaling pathway, and focal adhesion, have also been reported to be critical in the development of pregnancy (Gupta et al., 2016; Menon, 2016; Sharma et al., 2016). Our results revealed comprehensive details of the essential intercellular signal cascade during peripartum.

We further compared the intercellular communication occurring in the DBD and DAD. ECs were prominent receivers of differential signals from other cells in the DAD, mainly including Wnt and BMP-BMPR signaling. In contrast, EECs were the important receivers of cell signaling in the DBD, and Wnt signals also play an important role in intercellular crosstalk from other cells to EECs. Some studies reported that the Wnt signaling pathway is not just involved in early pregnancy but also takes part in the cascade events that lead to labor (Pereyra et al., 2019). Therefore, future studies on the Wnt signaling pathway are hoped to provide deeper insights into the pathophysiological significance of these proteins in pregnancy events. The diagnosis of labor onset has been described as one of the most difficult and important judgments made by providers of maternity care (Hanley et al., 2016). Our results also implicate these ligands/receptors of distinct intercellular communications as factors governing labor onset and, ultimately, candidate biomarkers for labor prediction.

T cells that populate the decidua have important roles in both normal and pathological pregnancies (Mincheva-Nilsson et al., 2000), but understanding the functions of T cells at the maternal-fetal interface remains one of the most difficult problems in reproductive immunology (Nancy and Erlebacher, 2014). We investigated the intercellular communication between TCs and other decidual cells and found that signals relayed from TCs to other cells were distinctly increased in the DAD, some of which, e.g. TNFSF14-TNFRSF14/LTBR and FASLG-FAS, are involved in mother-fetal immune tolerance and facilitating the onset of labor (Guller and Lachapelle, 1999; Guo C. et al., 2021). In comparison, the intercellular communications between TCs and other cells were noticeably decreased, indicating that, when delivery occurs, the maintenance of mother-fetal immune tolerance is no longer unnecessary in the uterine microenvironment; therefore, the chemokine network targeting T cells is shut down.

To further explore the dynamic changes to the maternal-fetal immune system during delivery, we investigated the characteristics of the BCR and TCR repertoires in the DBD and DAD by scBCR/TCR seq. The results showed that the frequency of clonal B cells increased and that of T cells decreased in the DAD. Although, the clonal diversity and evenness analysis of BCRs/TCRs showed no significant differences in the clonal expansion of B/T cells between the DAD and DBD. These results indicate that adaptive immunity does not significantly change at the maternal-fetal interface during normal labor. Accumulating evidence suggests that innate immune cells (neutrophils, macrophages, and mast cells) mediate the process of labor by releasing pro-inflammatory factors. However, adaptive immune cells (B/T cells) participate in the maintenance of feto-maternal tolerance during pregnancy, and alterations in their function or abundance may lead to labor at term or preterm (Gomez-Lopez et al., 2014). Therefore, all these results indicate that adaptive immunity must remain stable to maintain maternal-fetal tolerance via intercellular communication during normal labor.

In this report, we have described a comprehensive cell-cell communication network active in the peripartum decidua during delivery and found many common and differential intercellular signaling pathways among the different decidual cells between DBD and DAD, some of which represent candidate biomarkers for the diagnosis of labor. We further investigated the TC-related communications between the DBD and DAD and discovered that T cells may play key roles in full-term delivery. The results of scTCR/BCR-seq showed no significant differences in the clonal expansion of B/T cells between DAD and DBD, suggesting that adaptive immunity at the maternal-fetal interface does not change significantly during delivery. In summary, this study provided a comprehensive overview of the landscape of intercellular communication in the peripartum decidua and identified some key intercellular signals involved in labor and maternal-fetal immune tolerance. We believe that our study provides clues to understanding the mechanisms of pregnancy and possible diagnostic strategies for the onset of labor.

## Data Availability

The datasets presented in this study can be found in online repositories. The names of the repository/repositories and accession number(s) can be found below: GEO: GSE186368.
